# Crystal structure of hy­droxy scandium nitrate chloride

**DOI:** 10.1107/S2056989019003918

**Published:** 2019-04-02

**Authors:** Jeremiah Sears, Roger Cramer, Timothy Boyle

**Affiliations:** a Sandia National Laboratories, Advanced Materials Laboratory, 1001 University, Boulevard, SE, Albuquerque, NM 87106, USA; b University of Hawaii - Manoa, Department of Chemistry, 2545 McCarthy Mall, Honolulu, HI 96822-2275, USA

**Keywords:** scandium, hepta­coordinate, nitrate, crystal structure

## Abstract

Each Sc^3+^ ion in the title salt, di-μ-hydroxido-bis­[tri­aqua­(nitrato-κ^2^ *O*,*O*′)scandium(III)] dichloride, [Sc_2_(NO_3_)_2_(OH)_2_(H_2_O)_6_]Cl_2_, is coordinated by a nitrate anion, two hydroxide ions and three water mol­ecules to generate a distorted penta­gonal–bipyramidal ScO_7_ coordination polyhedron. The complete {[(NO_3_)(μ-OH)Sc(H_2_O)_3_]_2_}^2+^ ion is generated by crystallographic inversion symmetry. The nitrate anion binds in a bidentate fashion whereas the hydroxide ions are bridged between two Sc centers. Two charge-balancing Cl^−^ ions are located in the outer sphere. In the extended structure, O—H⋯O and O—H⋯Cl hydrogen bonds connect the components into a three-dimensional network.

## Abstract 2

Each Sc^3+^ ion in the title salt, [Sc_2_(NO_3_)_2_(OH)_2_(H_2_O)_6_]Cl_2_, is coordinated by a nitrate anion, two hydroxide ions and three water mol­ecules to generate a distorted penta­gonal–bipyramidal ScO_7_ coordination polyhedron. The complete {[(NO_3_)(μ-OH)Sc(H_2_O)_3_]_2_}^2+^ ion is generated by crystallographic inversion symmetry. The nitrate anion binds in a bidentate fashion whereas the hydroxide ions are bridged between two Sc centers.

## Chemical context   

Scandium nitrate compounds have found widespread utility in a diverse number of applications, including catalysts for aqueous-based organic reactions (Kobayashi, 1999 ▸), heterogeneous Lewis acid catalysts (Cao *et al.*, 2015 ▸), cyano­silylation catalysis (Zhang *et al.*, 2015 ▸) and films for use in optics and electronic manufacturing (Wang *et al.*, 2013 ▸). Previously, the structural properties of scandium salts were reviewed and the wide variety of structure types available for Sc metal were presented (Sears *et al.*, 2017 ▸). From this review, the diversity of structurally characterized scandium nitrate salts was illumin­ated. These were found to possess inner-sphere, outer-sphere and mixed-sphere nitrate ions. Additionally, a number of bridging ligands (OH^−^, OMe^−^) were present. As we continue to explore the fundamental coordination behavior of scandium with nitric acid as a means to recycle this multipurpose metal, another unusual scandium nitrate structure [(κ^2^-NO_3_)(μ-OH)Sc(H_2_O)_3_]_2_2(Cl) (**1**) was isolated. This report details the structure and its relationship to known scandium nitrate derivatives.
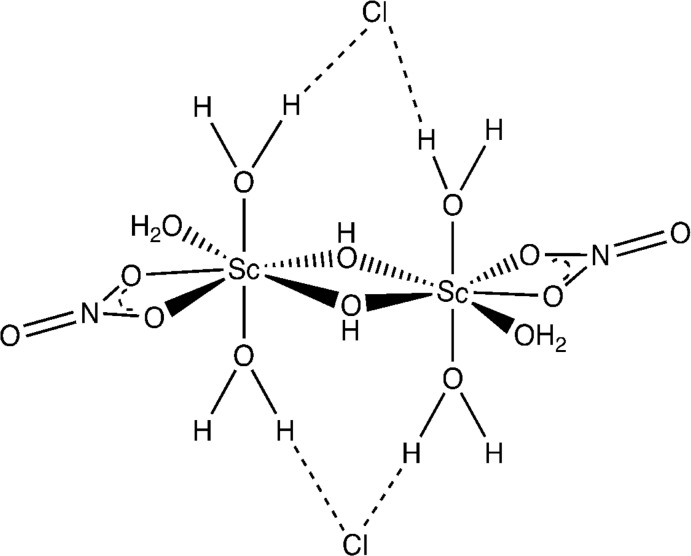


## Structural commentary   

**Figure 1:**
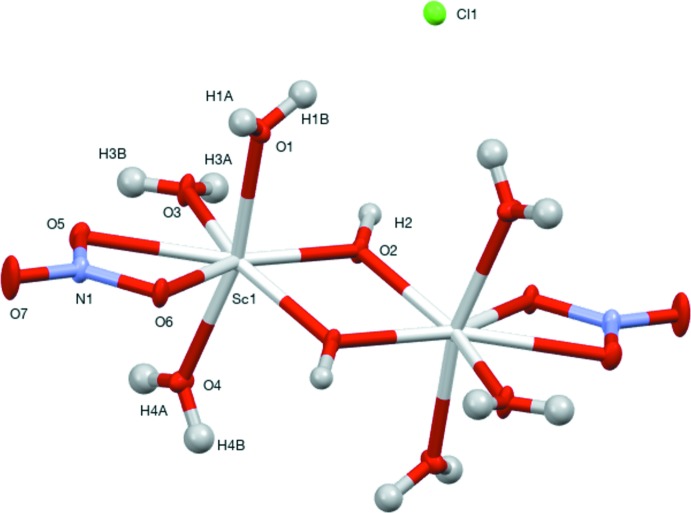
The mol­ecular structure of the title compound, with non-H atoms shown as displacement ellipsoids at the 50% probability level. Only one Cl^−^ ion is shown. Unlabeled atoms are generated by the symmetry operation 1 − *x*, −*y*, 1 − *z*.

The title compound (Fig. 1 ▸) is the third reported hydrated scandium nitrate salt. We previously isolated [(H_2_O)_4_Sc(*k* ^2^-NO_3_)_2_](NO_3_)H_2_O and [(H_2_O)_3_Sc(*k* ^2^-NO_3_)(μ-OH)]_2_2(NO_3_) from the reaction of [(H_2_O)_5_Sc(μ-OH)]_2_4(Cl)2(H_2_O) with concentrated nitric acid at elevated and room temperatures, respectively. Similarities between [(*k* ^2^-*NO_3_)*(μ-OH)Sc(H_2_O)_3_]_2_2(Cl) and [(H_2_O)_3_Sc(*k* ^2^-NO_3_)(μ-OH)]_2_2(NO_3_) were expected and observed.

The axial water mol­ecules are distorted from linearity more so for **1** [O1—Sc1—O4 = 166.48 (2)°] than for [(H_2_O)_3_Sc(*k* ^2^-NO_3_)(μ-OH)]_2_2(NO_3_) [O4—Sc—O6 = 171.52 (7)°]. The Sc—O (H_2_O) bond distances of 2.124 (1)–2.148 (1) Å for **1** are comparable to the 2.114 (2)–2.183 (1) Å distances reported for the other hydroxide-bridged NO_3_ salt. Bond angles between the axial water mol­ecules and the remaining nearly coplanar equatorial ligands range from 79.56 (2)–100.60 (2)° for **1** and 82.50 (6)–99.96 (6)° for [(H_2_O)_3_Sc(*k* ^2^-NO_3_)(μ-OH)]_2_2(NO_3_). The angles between the equatorial oxygen atoms range from 55.61 (2)–81.36 (2)° and 55.76 (5)–83.70 (6)°, respectively. The shortest Sc—O bond distances, 2.0542 (5)–2.0569 (5) Å for **1** and 2.053 (2)–2.076 (1) Å for [(H_2_O)_3_Sc(*k* ^2^-NO_3_)(μ-OH)]_2_2(NO_3_), occur for the bridging hydroxide ions. In both salts, the bidentate NO_3_ ions have the weakest inter­action with Sc—O bond distances of 2.291 (1)–2.314 (1) Å for **1** and 2.114 (2)–2.183 (1) Å for [(H_2_O)_3_Sc(*k* ^2^-NO_3_)(μ-OH)]_2_2(NO_3_).

The precursor to **1**, [(H_2_O)_5_Sc(μ-OH)]_2_4(Cl)2(H_2_O), is also a seven-coordinate Sc salt. Rotation of the precursor reveals a capped trigonal–prismatic geometry about the Sc centers that is useful for comparison. Equatorial ligand angles for [(H_2_O)_5_Sc(μ-OH)]_2_4(Cl)2(H_2_O) had a much smaller range of 83.32–95.17°. Dihedral angles between axial and equatorial ligands for the precursor also have a significantly reduced range of 77.18–79.09°. These differences further support the distorted penta­gonal–bipyramidal geometry assigned to **1**.

## Supra­molecular features   

**Figure 2:**
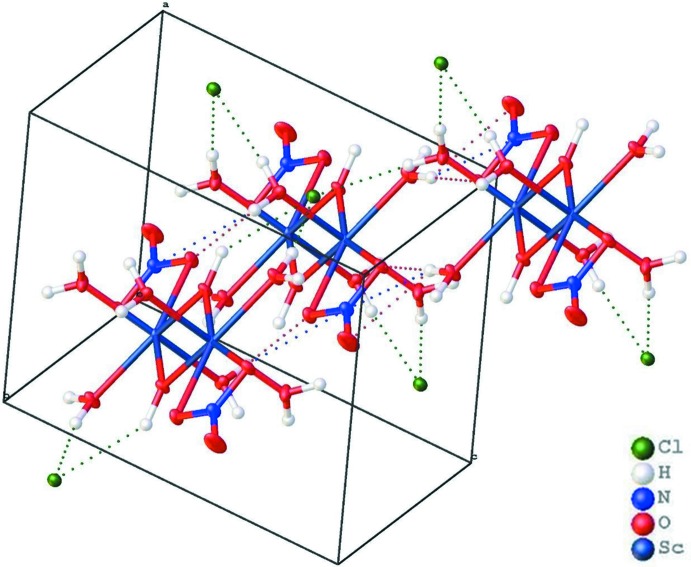
Partial packing diagram of the title compound, showing hydrogen bonds as dashed lines.

A network of scandium hy­droxy nitrate dimer chains that inter­act *via* separate equatorial coordinated water mol­ecules and nitrate ions with one another is observed for [(*k* ^2^-NO_3_)(μ-OH)Sc(H_2_O)_3_]_2_2(Cl). These chains are further linked into a three-dimensional network (Fig. 2 ▸) by O—H⋯Cl hydrogen bonds between axially as well as equatorially coordinated water mol­ecules and outer sphere Cl^−^ anions indicated by the symmetry operations in Table 1 ▸.

## Database survey   

There are two reports of hydrated scandium nitrates, [(H_2_O)_4_Sc(*k* ^2^-NO_3_)_2_](NO_3_)(H_2_O) (Boyle *et al.*, 2015 ▸) and [(H_2_O)_3_Sc(*k* ^2^-NO_3_)(μ-OH)]_2_2(NO_3_) (Wang *et al.*, 2013 ▸; Boyle *et al.* 2015 ▸), and both contain outer-sphere nitrate anions. As expected a similar network is observed for [(H_2_O)_3_Sc(*k* ^2^-NO_3_)(μ-OH)]_2_2(NO_3_). Salt **1** is the first reported hydrated scandium nitrate to contain outer-sphere chloride anions.

## Synthesis and crystallization   

Salt **1** was isolated from a cooled (273 K) mixture of [(H_2_O)_5_Sc(μ-OH)]_2_4(Cl)2(H_2_O) dissolved in water and an equal volume of concentrated HNO_3_(aq). The reaction was slowly warmed to room temperature and set aside for slow evaporation until crystals formed. From this mixture, a single crystal of **1** was selected and used for single crystal X-ray analysis. Note: Both [(H_2_O)_4_Sc(κ^2^-NO_3_)_2_]NO_3_(H_2_O) and [(H_2_O)_3_Sc(κ^2^-NO_3_)(μ-OH)]_2_2(NO_3_) have also been isolated from this preparatory route (Boyle *et al.*, 2015 ▸).

## Refinement   

Crystal data, data collection and structure refinement details are summarized in Table 2 ▸.

## Supplementary Material

Crystal structure: contains datablock(s) global, I. DOI: 10.1107/S2056989019003918/hb4231sup1.cif

Structure factors: contains datablock(s) I. DOI: 10.1107/S2056989019003918/hb4231Isup2.hkl

CCDC reference: 1904826

Additional supporting information:  crystallographic information; 3D view; checkCIF report

## Figures and Tables

**Table 1:** Hydrogen-bond geometry (Å, °)

*D*—H⋯*A*	*D*—H	H⋯*A*	*D*⋯*A*	*D*—H⋯*A*
O1—H1*A*⋯O6^i^	0.782 (16)	2.051 (16)	2.8175 (7)	166.6 (16)
O1—H1*B*⋯Cl1^ii^	0.787 (15)	2.300 (15)	3.0856 (6)	176.6 (14)
O2—H2⋯Cl1^iii^	0.692 (16)	2.617 (16)	3.2749 (5)	159.7 (17)
O3—H3*A*⋯Cl1^iii^	0.815 (16)	2.305 (16)	3.1017 (6)	165.9 (15)
O3—H3*B*⋯O5^iv^	0.811 (15)	1.989 (15)	2.7977 (7)	175.0 (14)
O4—H4*A*⋯Cl1^v^	0.859 (15)	2.316 (15)	3.1722 (6)	174.5 (13)
O4—H4*B*⋯Cl1	0.824 (16)	2.242 (16)	3.0658 (6)	179.0 (15)

Symmetry codes: (i) 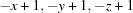; (ii) 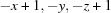; (iii) 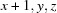; (iv) 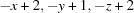; (v) 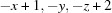.

**Table 2:** Experimental details

Crystal data	
Chemical formula	[Sc_2_(NO_3_)_2_(OH)_2_(H_2_O)_6_]2(Cl)
*M*_r_	426.95
Crystal system, space group	Triclinic, *P* 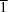
Temperature (K)	100
*a*, *b*, *c* (Å)	6.7221 (3), 7.6279 (4), 8.5181 (4)
α, β, γ (°)	100.904 (2), 110.125 (2), 102.329 (2)
*V* (Å^3^)	383.87 (3)
*Z*	1
Radiation type	Mo *K*α
μ (mm^−1^)	1.30
Crystal size (mm)	0.52 × 0.24 × 0.21

Data collection	
Diffractometer	Bruker APEXII CCD
Absorption correction	Multi-scan (*SADABS*; Bruker, 2016)
*T*_min_, *T*_max_	0.634, 0.749
No. of measured, independent and observed [*I* > 2σ(*I*)] reflections	21806, 5393, 4597
*R*_int_	0.025
(sin θ/λ)_max_ (Å^−1^)	0.944

Refinement	
*R*[*F* ^2^ > 2σ(*F* ^2^)], *wR*(*F* ^2^), *S*	0.024, 0.056, 1.04
No. of reflections	5393
No. of parameters	119
H-atom treatment	All H-atom parameters refined
Δρ_max_, Δρ_min_ (e Å^−3^)	0.50, −0.35

Computer programs: *APEX3* and *SAINT* (Bruker, 2016 ▸), *SHELXT* (Sheldrick, 2015*a* ▸), *SHELXL* (Sheldrick, 2015*b* ▸) and *OLEX2* (Dolomanov *et al.*, 2009 ▸).

## supplementary crystallographic information

### Crystal data

**Table:**

[Sc_2_(NO_3_)_2_(OH)_2_(H_2_O)_6_]2(Cl)	*Z* = 1
*M**_r_* = 426.95	*F*(000) = 216
Triclinic, *P*1	*D*_x_ = 1.847 Mg m^−^^3^
*a* = 6.7221 (3) Å	Mo *K*α radiation, λ = 0.71073 Å
*b* = 7.6279 (4) Å	Cell parameters from 9960 reflections
*c* = 8.5181 (4) Å	θ = 2.7–43.4°
α = 100.904 (2)°	µ = 1.30 mm^−^^1^
β = 110.125 (2)°	*T* = 100 K
γ = 102.329 (2)°	Plate, colourless
*V* = 383.87 (3) Å^3^	0.52 × 0.24 × 0.21 mm

### Data collection

**Table:**

Bruker APEXII CCD diffractometer	4597 reflections with *I* > 2σ(*I*)
Radiation source: fine–focus tube	*R*_int_ = 0.025
φ and ω scans	θ_max_ = 42.1°, θ_min_ = 2.7°
Absorption correction: multi-scan (SADABS; Bruker, 2016)	*h* = −12→12
*T*_min_ = 0.634, *T*_max_ = 0.749	*k* = −14→14
21806 measured reflections	*l* = −16→15
5393 independent reflections	

### Refinement

**Table:**

Refinement on *F*^2^	Primary atom site location: structure-invariant direct methods
Least-squares matrix: full	Hydrogen site location: difference Fourier map
*R*[*F*^2^ > 2σ(*F*^2^)] = 0.024	All H-atom parameters refined
*wR*(*F*^2^) = 0.056	*w* = 1/[σ^2^(*F*_o_^2^) + (0.026*P*)^2^ + 0.0527*P*] where *P* = (*F*_o_^2^ + 2*F*_c_^2^)/3
*S* = 1.04	(Δ/σ)_max_ = 0.001
5393 reflections	Δρ_max_ = 0.50 e Å^−^^3^
119 parameters	Δρ_min_ = −0.35 e Å^−^^3^
0 restraints	

### Special details

**Table:**

Geometry. All esds (except the esd in the dihedral angle between two l.s. planes) are estimated using the full covariance matrix. The cell esds are taken into account individually in the estimation of esds in distances, angles and torsion angles; correlations between esds in cell parameters are only used when they are defined by crystal symmetry. An approximate (isotropic) treatment of cell esds is used for estimating esds involving l.s. planes.

### Fractional atomic coordinates and isotropic or equivalent isotropic displacement parameters (Å^2^)

**Table:**

	*x*	*y*	*z*	*U*_iso_*/*U*_eq_	
Sc1	0.64421 (2)	0.20102 (2)	0.65362 (2)	0.00619 (2)	
O1	0.77577 (9)	0.34667 (7)	0.50248 (7)	0.01145 (8)	
H1A	0.730 (3)	0.425 (2)	0.469 (2)	0.037 (4)*	
H1B	0.814 (2)	0.297 (2)	0.4330 (19)	0.031 (4)*	
O2	0.66804 (8)	−0.04815 (7)	0.53126 (6)	0.00944 (8)	
H2	0.755 (3)	−0.084 (2)	0.562 (2)	0.042 (4)*	
O3	0.97436 (9)	0.22378 (8)	0.82658 (7)	0.01256 (9)	
H3A	1.025 (3)	0.136 (2)	0.832 (2)	0.036 (4)*	
H3B	1.042 (2)	0.306 (2)	0.919 (2)	0.030 (4)*	
O4	0.54719 (9)	0.11936 (8)	0.84788 (7)	0.01370 (9)	
H4A	0.639 (2)	0.131 (2)	0.951 (2)	0.032 (4)*	
H4B	0.425 (3)	0.048 (2)	0.829 (2)	0.037 (4)*	
O5	0.77362 (8)	0.48678 (7)	0.86375 (7)	0.01144 (8)	
O6	0.45946 (9)	0.42130 (7)	0.64820 (6)	0.01136 (8)	
O7	0.57811 (11)	0.68156 (9)	0.85879 (8)	0.02282 (13)	
N1	0.60275 (10)	0.53719 (8)	0.79388 (8)	0.01116 (9)	
Cl1	0.09630 (3)	−0.14716 (2)	0.78189 (2)	0.01058 (3)	

### Atomic displacement parameters (Å^2^)

**Table:**

	*U*^11^	*U*^22^	*U*^33^	*U*^12^	*U*^13^	*U*^23^
Sc1	0.00664 (4)	0.00485 (4)	0.00490 (4)	0.00172 (3)	0.00048 (3)	0.00005 (3)
O1	0.0153 (2)	0.0105 (2)	0.0118 (2)	0.00636 (17)	0.00710 (17)	0.00454 (16)
O2	0.00739 (17)	0.00762 (18)	0.00909 (18)	0.00341 (14)	−0.00061 (14)	−0.00082 (14)
O3	0.01049 (19)	0.00957 (19)	0.0107 (2)	0.00455 (16)	−0.00204 (16)	−0.00219 (16)
O4	0.0119 (2)	0.0173 (2)	0.00861 (19)	0.00022 (17)	0.00226 (16)	0.00464 (17)
O5	0.00943 (18)	0.00930 (19)	0.01084 (19)	0.00451 (15)	−0.00074 (15)	−0.00048 (15)
O6	0.01149 (19)	0.00946 (19)	0.00762 (18)	0.00380 (15)	−0.00085 (15)	−0.00164 (15)
O7	0.0229 (3)	0.0165 (3)	0.0196 (3)	0.0137 (2)	−0.0006 (2)	−0.0075 (2)
N1	0.0113 (2)	0.0094 (2)	0.0092 (2)	0.00476 (17)	0.00089 (17)	−0.00115 (17)
Cl1	0.01037 (6)	0.01118 (6)	0.01048 (6)	0.00477 (5)	0.00374 (5)	0.00283 (5)

### Geometric parameters (Å, º)

**Table:**

Sc1—O2	2.0542 (5)	O2—Sc1^i^	2.0569 (5)
Sc1—O2^i^	2.0569 (5)	O2—H2	0.692 (16)
Sc1—O4	2.1238 (6)	O3—H3A	0.815 (16)
Sc1—O1	2.1399 (5)	O3—H3B	0.811 (15)
Sc1—O3	2.1482 (5)	O4—H4A	0.859 (15)
Sc1—O6	2.2910 (5)	O4—H4B	0.824 (16)
Sc1—O5	2.3140 (5)	O5—N1	1.2752 (8)
Sc1—Sc1^i^	3.3085 (3)	O6—N1	1.2837 (8)
O1—H1A	0.782 (16)	O7—N1	1.2068 (8)
O1—H1B	0.787 (15)		
			
O2—Sc1—O2^i^	72.83 (2)	O4—Sc1—Sc1^i^	95.494 (17)
O2—Sc1—O4	99.79 (2)	O1—Sc1—Sc1^i^	97.895 (16)
O2^i^—Sc1—O4	89.10 (2)	O3—Sc1—Sc1^i^	117.158 (16)
O2—Sc1—O1	92.12 (2)	O6—Sc1—Sc1^i^	114.024 (14)
O2^i^—Sc1—O1	100.60 (2)	O5—Sc1—Sc1^i^	168.016 (14)
O4—Sc1—O1	166.48 (2)	Sc1—O1—H1A	121.1 (11)
O2—Sc1—O3	81.36 (2)	Sc1—O1—H1B	122.4 (10)
O2^i^—Sc1—O3	152.16 (2)	H1A—O1—H1B	107.7 (15)
O4—Sc1—O3	85.12 (2)	Sc1—O2—Sc1^i^	107.17 (2)
O1—Sc1—O3	90.41 (2)	Sc1—O2—H2	125.6 (14)
O2—Sc1—O6	148.997 (19)	Sc1^i^—O2—H2	125.0 (14)
O2^i^—Sc1—O6	78.391 (19)	Sc1—O3—H3A	124.5 (11)
O4—Sc1—O6	90.93 (2)	Sc1—O3—H3B	122.1 (10)
O1—Sc1—O6	81.93 (2)	H3A—O3—H3B	108.8 (14)
O3—Sc1—O6	128.81 (2)	Sc1—O4—H4A	124.1 (10)
O2—Sc1—O5	154.887 (19)	Sc1—O4—H4B	125.0 (11)
O2^i^—Sc1—O5	131.997 (19)	H4A—O4—H4B	108.9 (14)
O4—Sc1—O5	79.56 (2)	N1—O5—Sc1	94.62 (4)
O1—Sc1—O5	86.94 (2)	N1—O6—Sc1	95.45 (4)
O3—Sc1—O5	73.561 (19)	O7—N1—O5	122.93 (6)
O6—Sc1—O5	55.610 (18)	O7—N1—O6	122.90 (6)
O2—Sc1—Sc1^i^	36.441 (14)	O5—N1—O6	114.17 (5)
O2^i^—Sc1—Sc1^i^	36.388 (14)		
			
Sc1—O5—N1—O7	176.54 (7)	Sc1—O6—N1—O7	−176.50 (7)
Sc1—O5—N1—O6	−3.65 (6)	Sc1—O6—N1—O5	3.69 (6)

Symmetry code: (i) −*x*+1, −*y*, −*z*+1.

### Hydrogen-bond geometry (Å, º)

**Table:**

*D*—H···*A*	*D*—H	H···*A*	*D*···*A*	*D*—H···*A*
O1—H1*A*···O6^ii^	0.782 (16)	2.051 (16)	2.8175 (7)	166.6 (16)
O1—H1*B*···Cl1^i^	0.787 (15)	2.300 (15)	3.0856 (6)	176.6 (14)
O2—H2···Cl1^iii^	0.692 (16)	2.617 (16)	3.2749 (5)	159.7 (17)
O3—H3*A*···Cl1^iii^	0.815 (16)	2.305 (16)	3.1017 (6)	165.9 (15)
O3—H3*B*···O5^iv^	0.811 (15)	1.989 (15)	2.7977 (7)	175.0 (14)
O4—H4*A*···Cl1^v^	0.859 (15)	2.316 (15)	3.1722 (6)	174.5 (13)
O4—H4*B*···Cl1	0.824 (16)	2.242 (16)	3.0658 (6)	179.0 (15)

Symmetry codes: (i) −*x*+1, −*y*, −*z*+1; (ii) −*x*+1, −*y*+1, −*z*+1; (iii) *x*+1, *y*, *z*; (iv) −*x*+2, −*y*+1, −*z*+2; (v) −*x*+1, −*y*, −*z*+2.
